# Metagenomic Next-Generation Sequencing in Infectious Diseases: Clinical Applications, Translational Challenges, and Future Directions

**DOI:** 10.3390/diagnostics15161991

**Published:** 2025-08-08

**Authors:** Ayman Elbehiry, Adil Abalkhail

**Affiliations:** Department of Public Health, College of Applied Medical Sciences, Qassim University, P.O. Box 6666, Buraydah 51452, Saudi Arabia; ar.elbehiry@qu.edu.sa

**Keywords:** metagenomic next-generation sequencing, infectious disease diagnostics, antimicrobial resistance surveillance, public health

## Abstract

Metagenomic next-generation sequencing (mNGS) is transforming infectious disease diagnostics by enabling simultaneous, hypothesis-free detection of a broad array of pathogens—including bacteria, viruses, fungi, and parasites—directly from clinical specimens such as cerebrospinal fluid, blood, and bronchoalveolar lavage fluid. Unlike traditional culture and targeted molecular assays, mNGS serves as a powerful complementary approach, capable of identifying novel, fastidious, and polymicrobial infections while also characterizing antimicrobial resistance (AMR) genes. These advantages are particularly relevant in diagnostically challenging scenarios, such as infections in immunocompromised patients, sepsis, and culture-negative cases. Despite its potential, mNGS remains underutilized in clinical microbiology due to persistent gaps between its technical capabilities and routine diagnostic adoption. This review addresses key translational challenges that limit the broader implementation of mNGS, especially in resource-constrained and critical care settings. We provide a comprehensive overview of the entire workflow—from sample processing and host DNA depletion to sequencing platforms and downstream bioinformatics—and highlight sources of variability, including contamination, human DNA interference, and inconsistencies in resistance gene annotation. Additionally, we explore the ethical, legal, and privacy implications of host genomic data, as well as economic and regulatory obstacles hindering mNGS integration into standard clinical practice. To illustrate clinical relevance, we examine real-world evidence from large-scale trials such as MATESHIP, GRAIDS, DISQVER, and NGS-CAP. Finally, we outline future directions involving artificial intelligence, multi-omics integration, cloud-based analytics, and portable sequencing technologies for point-of-care diagnostics. By addressing both current limitations and emerging innovations, this review offers a translational framework for integrating mNGS into precision diagnostics and infection management across diverse healthcare environments.

## 1. Introduction

Infectious diseases remain a leading cause of global morbidity and mortality, accounting for more than five million deaths annually, including approximately 1.27 million attributed to antimicrobial-resistant (AMR) infections in 2019 [1,2,3]. Conventional diagnostic methods—such as culture, microscopy, serology, and polymerase chain reaction (PCR)—have long supported clinical microbiology but suffer from critical limitations. These include prolonged turnaround times, failure to detect non-culturable or fastidious organisms, poor performance in polymicrobial infections, and restricted utility when the causative pathogen is unknown [4,5]. In high-risk populations, such as critically ill and immunocompromised patients, diagnostic delays often lead to empiric broad-spectrum antibiotic use, escalating healthcare costs and contributing to suboptimal outcomes [6,7].

Metagenomic next-generation sequencing (mNGS) has emerged as a transformative tool in infectious disease diagnostics by enabling hypothesis-free detection of microbial DNA or RNA directly from clinical specimens. Although commonly referred to as “unbiased,” it is important to recognize that mNGS workflows are subject to various sources of bias introduced during sample preparation, library construction, and bioinformatic analysis, all of which can affect sensitivity and taxonomic resolution [8,9]. In central nervous system infections, mNGS has demonstrated diagnostic yields as high as 63%, compared to less than 30% for conventional approaches [10]. It has also proven valuable in identifying rare, novel, or co-infecting pathogens missed by standard tests, particularly in patients with encephalitis, sepsis, or unexplained febrile illness [6,10,11,12]. However, mNGS is best viewed as a complementary tool—not a replacement—for traditional diagnostics. When integrated with culture, PCR, and serological assays, it significantly enhances diagnostic accuracy, especially in complex or ambiguous cases.

Recent advancements in long-read sequencing technologies, particularly those developed by Oxford Nanopore Technologies, have enabled real-time, portable genomic testing at the point of care [13,14]. These platforms have been deployed in field settings for rapid diagnosis during outbreaks of Ebola, Zika, and SARS-CoV-2, underscoring their utility in decentralized healthcare delivery [15,16]. Studies from South Africa and Zambia demonstrate that nanopore-based targeted sequencing of sputum samples can rapidly detect *Mycobacterium tuberculosis* (*M. tuberculosis*) and drug resistance markers, with results available in just hours [17]. Such capabilities open the door to real-time infectious disease surveillance in low-resource settings without the need for centralized laboratory infrastructure [18].

Next-generation sequencing (NGS) technologies are increasingly central to AMR surveillance. Whole genome sequencing (WGS) enables simultaneous detection of resistance determinants and virulence factors, providing high-resolution data for outbreak tracking and infection control [19,20]. International initiatives such as the Global Antimicrobial Resistance Surveillance System (GLASS) and the 100K Pathogen Genome Project leverage NGS to monitor AMR trends across geographic and population boundaries [21,22]. In *M. tuberculosis*, WGS has shown high concordance with phenotypic susceptibility testing, supporting its use in predicting resistance to both first- and second-line therapies [23,24,25]. Metagenomic sequencing also enables real-time detection of plasmid-mediated resistance genes—such as *mcr-1* and *blaNDM-5*—that often go undetected by routine phenotypic methods [26,27].

Despite its promise, routine clinical adoption of NGS faces several technical and operational challenges. Sample processing workflows often require host DNA depletion to improve microbial signal in low-biomass specimens [28]. Bioinformatic pipelines must be standardized to ensure reproducibility and clinically relevant interpretation [6]. Regulatory frameworks—including those from the Clinical Laboratory Improvement Amendments (CLIA) and the College of American Pathologists (CAP)—are beginning to accommodate metagenomic assays, but validation procedures and reimbursement models remain inconsistent and underdeveloped [7]. Meanwhile, artificial intelligence (AI) and machine learning (ML) are being applied to automate taxonomic classification, AMR gene detection, and clinical reporting, reducing turnaround times and improving interpretability. Platforms such as IDSeq, PathoScope, and One Codex exemplify efforts to make metagenomics more accessible to diagnostic laboratories [29,30].

Looking ahead, the future of infectious disease diagnostics lies in integrating multi-omics and host–pathogen interactions. Emerging approaches—such as host transcriptome profiling and single-cell RNA sequencing—are showing promise in differentiating bacterial versus viral infections and predicting disease severity [31,32]. Combining host immune signatures with microbial sequencing data may enable real-time, precision-guided infectious disease management [33]. Furthermore, ultra-portable sequencing technologies capable of generating results within hours are being evaluated for use in emergency departments, border surveillance, and field hospitals [34,35]. Nevertheless, ethical considerations—including incidental findings, patient privacy, and disparities in access—must be addressed to ensure equitable and responsible implementation [36,37,38].

This narrative review provides a comprehensive and forward-looking synthesis of NGS applications in infectious disease diagnostics and management. It begins with an overview of fundamental principles and emerging methodologies, followed by detailed discussion of clinical applications in pathogen detection, AMR profiling, and outbreak surveillance. The review further explores the role of NGS in advancing personalized medicine and the integration of AI and ML into diagnostic workflows. In addition, we address ethical, regulatory, and reimbursement challenges and propose future research priorities to facilitate broader adoption of mNGS across diverse healthcare environments.

**Figure 1:**
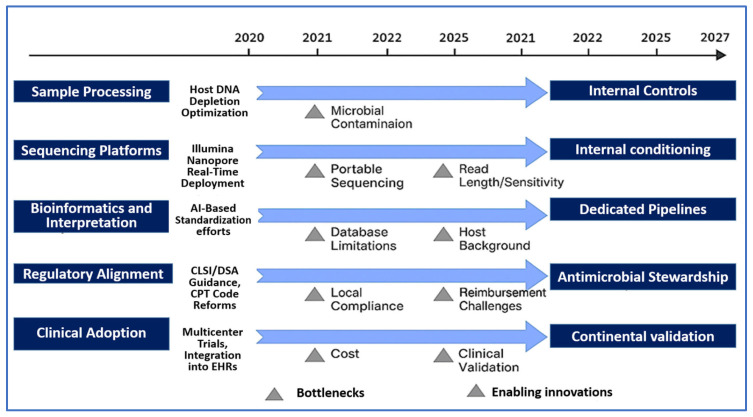
Translational Roadmap for Clinical mNGS Implementation. This schematic outlines a stepwise framework for translating mNGS from research settings into routine clinical diagnostics. The roadmap spans five interconnected domains with projected timelines: (1) advancements in sample preparation and host DNA depletion (2020–2023); (2) evolution of sequencing platforms, including the adoption of real-time and portable technologies (2019–2024); (3) standardization of bioinformatics pipelines and integration of artificial intelligence (2021–2025); (4) regulatory progress, including guidance from the Clinical and Laboratory Standards Institute (CLSI), Infectious Diseases Society of America (IDSA), and reforms in Current Procedural Terminology (CPT) coding (2022–2026); and (5) clinical adoption, encompassing multicenter validation trials, cost-effectiveness studies, and incorporation into electronic health records (2021–2027). The figure highlights key implementation barriers—such as host DNA contamination, inconsistent databases, and reimbursement delays—alongside enabling innovations, including AI-driven analytics, cloud-based workflows, and portable sequencing. Collectively, this roadmap supports a coordinated, multi-stakeholder strategy to guide the clinical integration of mNGS and advance precision diagnostics in infectious disease medicine.

To guide this transition from research to routine care, Figure 1 presents a translational roadmap for mNGS implementation. The schematic outlines key milestones and bottlenecks across five critical domains: sample preparation, sequencing platforms, bioinformatics, regulatory alignment, and clinical adoption. By mapping known barriers—such as host DNA contamination, database inconsistency, and reimbursement delays—alongside enabling innovations like AI and portable sequencing, the figure emphasizes a stepwise, systems-level strategy to integrate mNGS into clinical practice.

This review was developed based on a systematic literature search using PubMed, Scopus, Web of Science, and Google Scholar. Search terms included “metagenomic next-generation sequencing,” “mNGS,” “infectious disease diagnostics,” “antimicrobial resistance,” and “NGS in low-resource settings.” Articles published between 2010 and 2025 were screened for relevance. Priority was given to peer-reviewed original research, multicenter clinical trials, consensus guidelines, and real-world implementation studies.

## 2. Overview of NGS Technologies in Clinical Microbiology

NGS refers to a suite of high-throughput technologies capable of simultaneously analyzing millions of DNA or RNA fragments in parallel [39,40,41]. Unlike first-generation methods such as Sanger sequencing—which are limited by low throughput and the requirement for known target sequences—NGS allows for comprehensive, hypothesis-free genomic interrogation of clinical specimens [42]. This transformative capability has expanded the diagnostic landscape in clinical microbiology by enabling rapid identification of pathogens, detection of AMR genes, and real-time outbreak surveillance [4,5]. A variety of NGS platforms have been adopted in clinical settings, each offering unique strengths and limitations. WGS, which provides complete genomic coverage of cultured isolates, has become the gold standard for high-resolution bacterial typing. It enables precise taxonomic classification, phylogenetic tracking of outbreaks, and detection of both resistance and virulence determinants [22,43]. WGS is now routinely employed in global public health surveillance programs such as PulseNet and the Global GLASS.

The mNGS, by contrast, enables culture-independent sequencing of all nucleic acids within a clinical sample. This untargeted approach is particularly valuable in cases of unknown etiology, polymicrobial infections, or fastidious organisms [8,9]. However, clinical implementation of mNGS is challenged by the high abundance of host-derived nucleic acids, longer turnaround times, and the need for robust, standardized bioinformatics pipelines [7,10]. Targeted NGS panels offer an intermediate approach. These assays focus on predefined microbial or resistance gene targets using multiplex amplification or hybrid capture techniques. While more limited in scope than mNGS, targeted panels are faster, more cost-effective, and easier to interpret—making them highly suitable for syndromic testing of respiratory, bloodstream, or gastrointestinal infections [28,44]. Long-read sequencing platforms, such as those developed by Oxford Nanopore Technologies (ONT) and Pacific Biosciences (PacBio), provide a complementary dimension to short-read platforms. Capable of generating reads that span thousands of bases, these systems facilitate the resolution of repetitive regions, detection of structural variants, and complete reconstruction of plasmids and viral genomes [13,14]. ONT devices—including the portable MinION and Flongle—have enabled point-of-care diagnostics in resource-limited and outbreak-prone settings [16,34].

Emerging innovations such as host transcriptome and single-cell RNA sequencing are being explored to assess host immune responses and disease severity. These methods integrate pathogen detection with host biomarker profiling and may enable more refined diagnostic stratification and prognostication [31,33]. Additional advances—such as CRISPR-based enrichment, metatranscriptomics, and shotgun proteogenomics—are under investigation for their potential to improve sensitivity and strain-level resolution in clinical metagenomics [45]. Automation is another key driver of clinical NGS adoption. Integrated systems now combine nucleic acid extraction, library preparation, sequencing, and informatics into streamlined workflows that can deliver same-day results with minimal hands-on time [46]. These platforms have accelerated the use of NGS for common infectious syndromes such as pneumonia, bloodstream infections, and febrile illness of unknown origin.

Advances in bioinformatics are also improving the speed and accessibility of NGS interpretation. Cloud-based analysis tools such as IDSeq, One Codex, and CosmosID offer user-friendly interfaces, curated reference databases, and automated pipelines for taxonomic classification and resistance gene annotation [29,30]. These platforms have lowered the entry barrier for clinical laboratories without in-house bioinformatics infrastructure. As sequencing costs decline and analytic pipelines become increasingly standardized, NGS is evolving from a specialized research tool into a routine component of infectious disease diagnostics. Selecting the most appropriate NGS modality requires careful consideration of clinical indication, specimen type, turnaround requirements, and institutional resources. A comparative overview of major NGS modalities used in clinical microbiology is provided in Table 1. This summary highlights the sequencing scope, advantages, limitations, and representative clinical applications for WGS, mNGS, targeted panels, long-read sequencing, host transcriptomics, and automated platforms. Key literature supporting the clinical utility of each technology is also included to aid decision-making.

## 3. Clinical Applications of NGS in Infectious Disease Diagnosis and Management

NGS has fundamentally transformed infectious disease diagnostics by offering unparalleled resolution, speed, and breadth in identifying pathogens, profiling AMR, and informing clinical decision-making [47,48,49]. Unlike traditional methods that rely on targeted assays or culture-based techniques, NGS provides hypothesis-free, high-throughput analysis of microbial genomes, vastly enhancing diagnostic scope. This section highlights key translational applications of NGS in clinical microbiology, including its utility in diagnosing elusive infections, monitoring AMR, supporting outbreak investigations, and enabling precision-guided therapy. By incorporating NGS into clinical workflows, healthcare providers can deliver timely and targeted interventions—especially crucial in complex, immunocompromised, or critically ill patient populations.

### 3.1. Unbiased Pathogen Detection in Undiagnosed Cases

The mNGS has revolutionized pathogen detection by enabling unbiased identification of all microbial DNA or RNA present in clinical specimens [50,51]. Unlike conventional diagnostics—such as culture, serology, or PCR—that depend on prior assumptions, mNGS can simultaneously detect bacteria, viruses, fungi, and parasites in a single assay, without needing foreknowledge of the causative agent [4,8]. This approach has shown particular value in central nervous system (CNS) infections. In a seminal study, Wilson et al. [8], used mNGS on cerebrospinal fluid (CSF) to detect pathogens in 11% of previously undiagnosed cases of encephalitis and meningitis, including rare agents like *Taenia solium* and *Leptospira interrogans* that were missed by standard methods. More recently, Benoit et al. [52] reported a diagnostic sensitivity of 63.1% and specificity of 99.6% for CNS infections using mNGS, with particularly strong performance in immunocompromised and recently traveled patients.

Beyond CNS infections, mNGS has demonstrated remarkable utility in sepsis and critical care contexts. In a retrospective study of 130 intensive care unit (ICU) patients with sepsis, Chen et al. [53] found that mNGS achieved a detection rate of 87.7%, significantly outperforming conventional cultures (52.3%). Importantly, mNGS-guided modifications to antimicrobial therapy occurred in 72.3% of patients, contributing to early clinical improvement. Zuo et al. [54] further confirmed these findings in a prospective cohort of 277 ICU patients, showing mNGS sensitivity of 90.5% versus 36.0% with blood cultures. Interestingly, high microbial read counts also correlated with increased 30-day mortality, indicating a possible prognostic application.

Fever of unknown origin (FUO) is another diagnostic challenge. Lai et al. [55], in a retrospective study of 263 patients, found that mNGS yielded a diagnostic sensitivity of 81.5%—significantly higher than the 47.3% achieved by culture. Notably, mNGS results directly influenced treatment in nearly half of the cases. Similarly, in patients with severe community-acquired pneumonia, mNGS has shown impressive accuracy. Zhao et al. [56] reported that bronchoalveolar lavage fluid (BALF) analysis using mNGS identified pathogens in 95% of cases, compared to just 59% with standard diagnostics. In immunocompromised patients, mNGS-guided therapy reduced 28-day mortality from 74% to 31%.

Although blood cultures remain essential, mNGS has proven beneficial in detecting pathogens—especially viral and fungal—that often evade standard methods. For example, Qin et al. [57] found that mNGS identified pathogens in 77.7% of bloodstream infection cases versus 47.9% with blood culture. However, only 38.7% of detections overlapped between the methods, highlighting differing sensitivities and the ability of mNGS to detect DNA from non-viable or transient organisms. Thus, combined use of mNGS and traditional diagnostics offers the most comprehensive detection strategy. To further improve accuracy, Charalampous et al. [28] advocate for optimized host depletion and microbial enrichment techniques. In summary, mNGS provides substantial diagnostic advantages in undiagnosed infections, enabling timely and tailored clinical decisions. While interpretive complexities remain—particularly in distinguishing contamination from true infection—mNGS offers a vital complement to conventional diagnostics in critically ill or diagnostically complex cases.

### 3.2. Detection of Fastidious, Novel, and Polymicrobial Infections

One of the most compelling strengths of mNGS lies in its ability to detect fastidious, novel, and polymicrobial pathogens that often elude conventional diagnostics such as culture and PCR. Many clinically relevant microorganisms—such as *Bartonella* spp., *Coxiella burnetii*, and *Tropheryma whipplei*—are notoriously difficult to culture due to their intracellular localization or complex nutritional requirements. These organisms are frequently implicated in culture-negative endocarditis and undiagnosed febrile illnesses. In a representative case series, Wang et al. [58] applied mNGS to both blood and excised heart valve tissues from 23 patients with suspected culture-negative endocarditis. The assay identified *C. burnetii* in 21 cases and *Bartonella* species in 2, enabling targeted antimicrobial therapy and clinical recovery in over 90% of patients. Likewise, Lin et al. [59] detected *T. whipplei* via mNGS in BALF from immunocompromised patients with pneumonia—a rare and diagnostically challenging finding that had been missed by routine tests.

mNGS also plays a pivotal role in the detection of emerging and previously unknown pathogens. For example, during a hemorrhagic fever outbreak in the Democratic Republic of Congo, Briese et al. [60] used mNGS to identify a novel rhabdovirus, later named Bas-Congo virus, directly from patient specimens. Similarly, Grubaugh et al. [61] leveraged metagenomic sequencing to trace the cryptic emergence of Zika virus in Brazil, revealing months of undetected transmission prior to clinical recognition. These studies underscore mNGS’s power for early outbreak detection and global pathogen surveillance.

Polymicrobial infections—frequently underdiagnosed with conventional methods—represent another area where mNGS excels. Traditional cultures often isolate only the dominant or fast-growing organism, missing co-infections. In a large study by Zhao et al. [56], mNGS of BALF samples from ICU patients with severe community-acquired pneumonia detected mixed infections in 58.8% of cases, compared to just 18.0% identified by standard diagnostics. These findings led to tailored therapy adjustments and were associated with significantly improved 28-day survival. Integrative approaches further enhance diagnostic performance. Langelier et al. [62] combined host transcriptomics with microbial mNGS in tracheal aspirates from ICU patients with acute respiratory failure. This method not only differentiated infectious from non-infectious causes with high accuracy (AUC 0.96–0.88) but also uncovered co-infections and revealed host–pathogen interactions that informed clinical management.

Despite these strengths, mNGS is not without challenges. Low-abundance reads, background contamination, and sequencing artifacts can obscure clinically relevant signals—particularly in low-biomass samples. Salzberg et al. [6] highlighted the need for rigorous filtering criteria and clinically informed interpretive thresholds to reduce false positives and enhance diagnostic precision. In conclusion, mNGS represents a paradigm shift in detecting elusive, rare, or polymicrobial infections. Its ability to uncover fastidious and novel pathogens, often overlooked by traditional methods, makes it an invaluable tool in the diagnostic workup of complex, immunocompromised, or treatment-refractory patients.

### 3.3. AMR Profiling and Resistance Gene Surveillance

NGS has dramatically enhanced the surveillance of AMR by enabling comprehensive, high-resolution detection of resistance determinants—both chromosomally encoded and plasmid-mediated. WGS of bacterial isolates allows precise characterization of point mutations in resistance-associated genes (e.g., *gyrA*, *parC*, *rpoB*) and the identification of acquired resistance elements such as *blaKPC*, *mecA*, and *vanA* [63,64]. This information provides valuable insights into clonal transmission, outbreak dynamics, and resistance evolution. WGS is now routinely employed by leading public health agencies, including the CDC’s Antibiotic Resistance Laboratory Network and the WHO’s GLASS, to monitor resistance trends and inform infection control strategies [65,66]. The mNGS expands these capabilities to uncultured or polymicrobial clinical samples, enabling detection of resistance genes even when pathogens are non-viable or present at low abundance [67]. This is particularly advantageous in samples such as sputum, BALF, or cerebrospinal fluid, where culture may fail or take too long. For instance, mNGS can simultaneously identify pathogens and associated resistance determinants, accelerating time-to-therapy and improving clinical decision-making [28].

Recent advances in long-read nanopore sequencing have further accelerated AMR detection. Charalampous et al. [28] demonstrated that targeted nanopore sequencing of respiratory samples could detect both pathogens and resistance genes within six hours. Similarly, Votintseva et al. [35] applied nanopore sequencing directly to *M. tuberculosis*-positive sputum samples, accurately predicting resistance to rifampicin and isoniazid. These results support the use of rapid sequencing platforms for same-day diagnostics in tuberculosis and other high-priority infections. Enhanced sensitivity has also been achieved through novel enrichment methods. Gu et al. [9] developed a Cas9-mediated targeted enrichment strategy coupled with nanopore sequencing, enabling accurate detection of low-abundance resistance genes such as *blaCTX-M*, *tetM*, and *ermB*. This approach not only reduced sequencing noise from host DNA but also improved the resolution of resistome profiling in samples with complex microbial backgrounds.

Despite these advances, interpreting genotypic resistance remains a nuanced challenge. The presence of a resistance gene does not always equate to phenotypic resistance due to factors such as gene regulation, expression variability, and post-translational modifications [20]. This genotypic–phenotypic discordance may stem from several biological mechanisms, including (1) mutations in regulatory elements that suppress gene expression, (2) gene silencing by insertion sequences or repressor proteins, (3) absence of required co-factors or efflux pumps for resistance expression, and (4) post-translational modifications affecting protein function [68,69]. Additionally, resistance genes may be present on non-replicating plasmids or in subpopulations below detection thresholds for phenotypic assays [64]. In some cases, the resistance gene may be functional but not expressed under in vitro testing conditions, leading to false-negative phenotypic results [20].

To enhance interpretability, results from mNGS and WGS should be contextualized using curated resistance gene databases such as CARD, ResFinder, and MEGARes [70,71]. Integration with institutional antibiograms and clinical context remains essential for determining therapeutic relevance. In summary, NGS technologies—especially mNGS and WGS—are redefining AMR detection by offering rapid, comprehensive, and culture-independent resistance profiling. While challenges remain in translating genotypic findings into therapeutic action, continued improvements in sequencing platforms, enrichment techniques, and interpretive frameworks are propelling NGS-based AMR surveillance toward routine clinical use and antimicrobial stewardship.

### 3.4. NGS for Outbreak Investigation and Epidemiological Typing

NGS has emerged as a cornerstone technology for outbreak investigation and molecular epidemiology, offering unprecedented resolution in tracking pathogen transmission, differentiating clonal from unrelated strains, and identifying infection sources. WGS, in particular, provides single nucleotide polymorphism (SNP)-level resolution, far surpassing traditional typing methods such as pulsed-field gel electrophoresis (PFGE) or multilocus sequence typing (MLST) [49]. In a landmark investigation, Gorrie et al. [72] used WGS to trace the spread of multidrug-resistant *Klebsiella pneumoniae* (*K. pneumoniae*) in an ICU, uncovering unexpected environmental reservoirs and asymptomatic carriers that conventional methods had failed to detect. Similarly, Eyre et al. [73] demonstrated that only 35% of *Clostridioides difficile* infections in a hospital setting were attributable to transmission from symptomatic patients, challenging long-standing assumptions about nosocomial spread and prompting changes in infection control protocols.

Real-time WGS implementation has also demonstrated tangible clinical and economic impact. Sundermann et al. [74] conducted a 22-month prospective surveillance study at the University of Pittsburgh Medical Center involving over 3900 bacterial isolates. They identified 172 silent transmission clusters, prompting targeted interventions that interrupted 95.6% of transmission events. These actions prevented an estimated 62 infections and 4.8 deaths, while generating cost savings exceeding USD 695,000—highlighting the value of genomics-informed infection control.

WGS has also been instrumental in outbreak resolution beyond hospital settings. In a study by Silvotti et al. [75], WGS was employed to investigate a suspected outbreak of carbapenem-resistant *Klebsiella pneumoniae* in a neurorehabilitation unit in Italy. Genomic sequencing of 19 isolates revealed seven independent introductions of KPC-producing strains into the unit, with only two limited transmission chains. This unexpected finding shifted infection control efforts from widespread environmental decontamination to more targeted measures, including modifying staff workflows and patient placement protocols—ultimately leading to successful outbreak containment.

The utility of WGS extends far beyond local hospital surveillance. In global health, genome-based phylogenetics has played a pivotal role in tracking major viral epidemics, including the Ebola outbreak in West Africa [76] and the global spread of SARS-CoV-2 [77]. NGS enables near real-time monitoring of pathogen evolution, the emergence of novel variants, and geographic dissemination patterns—thereby empowering health authorities to implement timely and evidence-based responses. Collectively, these examples underscore the indispensable role of NGS in outbreak investigations, supporting both localized infection control and broader public health policy.

### 3.5. NGS-Guided Personalized Infectious Disease Management

NGS—particularly mNGS—has opened a new frontier in precision infectious disease management. It facilitates pathogen-directed therapy, antimicrobial de-escalation, and improved outcomes in critically ill, undiagnosed, or treatment-refractory patients. Its integration into clinical workflows allows for individualized treatment decisions, especially in scenarios where conventional diagnostics fail. In a multicenter retrospective cohort study, Zhao et al. [78] demonstrated that mNGS-guided therapy significantly improved outcomes in ICU patients with severe community-acquired pneumonia who were unresponsive to empirical antibiotics. Among 303 patients, mNGS performed on BALF samples led to antimicrobial regimen modification in 73.2% of cases, resulting in a marked reduction in 28-day mortality (28.0% vs. 43.9%)**,** shorter ICU stays, and higher clinical cure rates—highlighting the clinical utility of mNGS in tailoring treatment.

Lai et al. [79] analyzed 400 patients with suspected lower respiratory tract infections unresponsive to empirical therapy. mNGS of BALF identified pathogens in 93.3% of cases, compared to only 55.6% by conventional diagnostics. Treatment was adjusted in over half of the cases, with 60.8% of patients showing clinical improvement, reinforcing mNGS’s role in guiding effective, targeted interventions. In immunocompromised patients, the diagnostic advantage of mNGS is particularly pronounced. Zheng et al. [80] evaluated BALF samples from patients with community-acquired pneumonia and found that mNGS achieved a diagnostic yield of 79.1%, far exceeding that of culture (16.0%). Mixed infections were identified in 47.6% of patients. Antimicrobial regimens were adjusted in 73.2% of cases, leading to clinical improvement in over half and confirming treatment adequacy in an additional 22.6%. These data highlight mNGS as a critical tool in managing complex infections in vulnerable populations.

Beyond respiratory diseases, mNGS has shown excellent diagnostic accuracy in periprosthetic joint infections (PJIs), where culture-based methods often fail. Cai et al. [81] assessed 44 suspected PJI cases and found that mNGS on periprosthetic tissue achieved 95.5% sensitivity and 90.9% specificity, outperforming culture (72.7% and 77.3%, respectively). This superior detection enabled more targeted therapy, reduced treatment duration, and lowered hospital costs—demonstrating the economic and clinical advantages of mNGS in orthopedic infections.

### 3.6. Real-World Clinical Impact of mNGS Across Diverse Infectious Syndromes

The integration of mNGS into clinical practice has transformed the landscape of infectious disease diagnostics. One of its most compelling strengths is its real-world applicability across a wide spectrum of infectious syndromes and patient populations. In a prospective study by Xiang et al. [82], 113 adult patients with severe pneumonia following cardiac surgery were assessed. Pathogen identification via mNGS of BALF achieved a diagnostic yield of 98.2%, compared to only 58.4% with standard microbiological testing. mNGS-guided therapy led to improved SOFA scores, reduced mechanical ventilation durations, and shorter ICU stays—underscoring its profound impact on precision clinical management.

Spinal infections are another area where mNGS has shown exceptional utility. Shi et al. [83] evaluated biopsy samples from 162 patients with suspected vertebral osteomyelitis and found that mNGS detected pathogens in 77.8% of cases, compared to just 27.2% by culture. Importantly, it identified anaerobic and polymicrobial infections often missed by traditional methods and maintained a high specificity (90.3%) even in patients previously exposed to broad-spectrum antimicrobials.

In PJIs, mNGS has consistently outperformed conventional diagnostics. Huang et al. [84] reported that mNGS of synovial fluid yielded a sensitivity of 89% and specificity of 95%**,** allowing clinicians to de-escalate antimicrobial therapy without compromising efficacy. Similarly, Shi et al. [85] demonstrated comparable performance (sensitivity 89.1%, specificity 94.7%) and emphasized mNGS’s ability to detect fastidious and rare pathogens such as *Cutibacterium acnes*, *Granulicatella adiacens*, and *M. tuberculosis* complex—supporting more precise and confident therapy decisions.

mNGS has also shown great promise in systemic and bloodstream infections. In a multicenter cohort study, Zhang et al. [86] analyzed plasma cell-free DNA from 212 patients with suspected systemic infections. mNGS detected pathogens in 74.4% of cases, far outperforming blood culture (12.1%). Antimicrobial management was modified in 70.3% of cases, and earlier mNGS testing was correlated with shorter hospital stays—further supporting its value in hard-to-diagnose infections.

**Figure 2:**
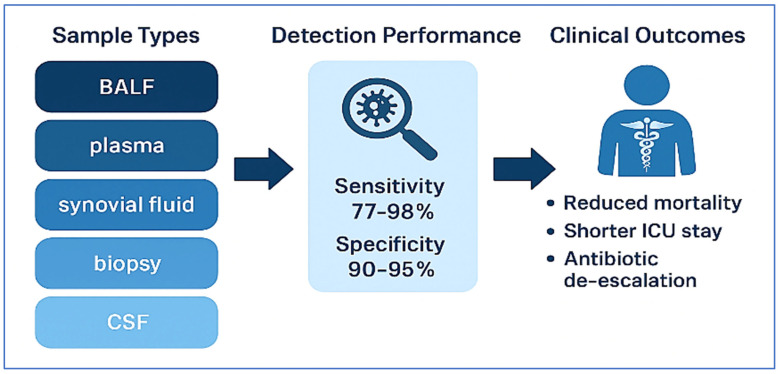
Clinical impact of mNGS across infectious syndromes: from diagnostic yield to tailored therapy. This flowchart illustrates the integration of mNGS into clinical workflows across a variety of infectious disease contexts and specimen types—including BALF, plasma, CSF, synovial fluid, and tissue biopsies. It highlights the diagnostic performance of mNGS, with reported sensitivity and specificity ranges, and maps its downstream clinical benefits. These include enhanced pathogen detection, antimicrobial de-escalation, reduced ICU length of stay, decreased mortality rates, and more precise, personalized treatment strategies—underscoring the transformative role of mNGS in modern infectious disease management.

Together, these studies establish mNGS as a revolutionary diagnostic tool in modern infectious disease management. Its ability to deliver rapid, accurate, and comprehensive pathogen identification translates into more targeted therapies, reduced antimicrobial misuse, and improved clinical outcomes. As sequencing costs decline and bioinformatics tools become more streamlined, mNGS is poised to become a routine component of diagnostic workflows in high-resource healthcare systems. To visually contextualize the clinical value of mNGS, Figure 2 summarizes its applications across diverse infectious syndromes. The diagram outlines sample types, diagnostic performance (sensitivity and specificity), and the downstream clinical impact—including mortality reduction, optimized antibiotic use, and shorter hospitalizations.

To facilitate a comparative understanding of the clinical applications, diagnostic performance, and therapeutic impact of mNGS across a range of infectious syndromes, Table 2 presents representative real-world studies that underscore its translational utility. These examples illustrate the breadth of mNGS’s clinical relevance—from improved pathogen detection to tailored antimicrobial therapy and favorable patient outcomes. Despite the growing enthusiasm surrounding mNGS, the lack of standardized data interpretation remains a significant barrier to its widespread clinical implementation. In recognition of this need, professional organizations—including the CAP, the IDSA, and the CLSI—have issued guidance on key aspects of mNGS validation and reporting. These include analytical performance benchmarks, contamination filtering strategies, taxonomic classification standards, and frameworks for clinical result interpretation.

Such initiatives aim to harmonize bioinformatics pipelines, define actionable reporting thresholds, and establish best practices for integrating mNGS into diagnostic workflows. By enhancing diagnostic reproducibility and clinical confidence, these standardized approaches are essential for the safe, effective, and scalable adoption of mNGS in routine care. As utilization continues to expand, adherence to these guidelines will be pivotal in unlocking the full clinical value of mNGS technologies across diverse healthcare settings.

## 4. Translational Challenges and Future Directions in NGS-Based Infectious Disease Diagnostics

**Figure 3:**
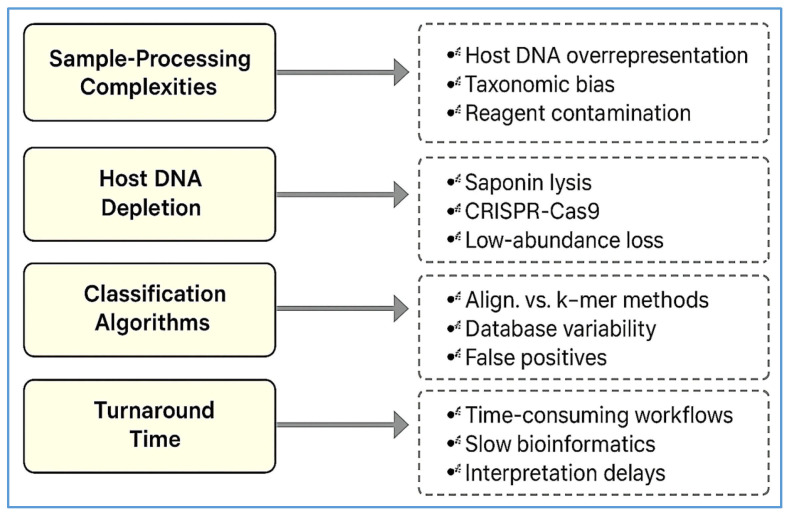
A visual summary of the major technical limitations affecting each stage of clinical mNGS. The flowchart is organized into three tiers: (1) sample processing challenges, including host DNA overrepresentation, reagent contamination, and lysis bias; (2) sequencing platform limitations, such as error rates, instrument variability, and library preparation bias; and (3) bioinformatics hurdles, including classifier algorithm inconsistencies, database incompleteness, and lack of interpretive standards. This framework complements Figure 1 by focusing exclusively on granular, micro-level bottlenecks that influence the diagnostic accuracy, sensitivity, and clinical utility of mNGS-based assays.

Despite the transformative potential of NGS—and mNGS in particular—its widespread clinical adoption remains constrained by a range of technical, operational, and ethical challenges. Overcoming these limitations is critical to advancing mNGS from an innovative research platform to a standardized component of diagnostic microbiology, precision medicine, and public health surveillance. To elucidate the specific bottlenecks that hinder clinical implementation, Figure 3 provides a tiered schematic of translational barriers encountered across the mNGS diagnostic pipeline. These challenges are grouped into three major categories: sample processing, sequencing technology, and bioinformatics interpretation. This granular breakdown complements the broader translational framework presented in Figure 1, offering a micro-level perspective on the practical and technical issues that must be addressed to ensure accurate, reproducible, and clinically actionable mNGS results.

### 4.1. Technical Barriers and Sample-Processing Complexities

One of the most persistent technical obstacles in mNGS diagnostics is the overwhelming presence of host-derived DNA in clinical specimens. In plasma, CSF, and BALF, human DNA can constitute over 90% of sequencing reads, significantly diluting microbial signal and reducing the sensitivity of pathogen detection—particularly in low-biomass infections [87]. Marotz et al. [88] observed that microbial cell-free DNA (cfDNA) often accounts for less than 5% of total reads across diverse specimen types, including saliva, skin, nasal, and vaginal swabs.

To overcome this imbalance, several host DNA depletion methods have been developed. Saponin-mediated lysis followed by DNase digestion is among the most commonly used and can reduce host DNA content by over 90%, improving microbial read recovery up to 20-fold in BALF [89]. However, this technique may introduce taxonomic bias due to differential lysis susceptibility [88]. Gram-negative bacteria, with their robust outer membranes and less permeable cell walls, are more resistant to saponin-mediated lysis and DNase digestion, leading to underrepresentation in sequencing results compared to more vulnerable Gram-positive organisms [51,88].

Alternative strategies such as CRISPR-Cas9-based depletion methods—e.g., Depletion of Abundant Sequences by Hybridization (DASH)—enable targeted removal of mitochondrial and ribosomal host sequences, achieving up to 99% host DNA reduction [90]. While highly effective, these methods remain complex, costly, and currently impractical for widespread clinical use. Mechanical lysis techniques like bead-beating improve recovery from hard-to-lyse organisms, such as fungi and mycobacteria, but risk nucleic acid fragmentation and inter-sample variability without strict standardization [28].

Importantly, host depletion methods involve tradeoffs. A multicenter evaluation showed that while microbial signal-to-noise ratios improved, pathogen detection sensitivity dropped when organism loads fell below 500 CFU/mL—due to inadvertent loss of target DNA during processing [91]. Such losses may lead to diagnostic delays or inappropriate therapy, underscoring the need to balance depletion efficacy with microbial recovery.

Environmental and reagent-derived DNA contamination—commonly referred to as the “kitome”—poses another major technical hurdle. DNA residues from ultrapure water, reagents, and plasticware can contribute spurious reads, particularly in low-biomass samples [92]. Contaminants have been shown to dominate up to 45% of sequencing reads in some samples [93]. Moreover, cross-contamination during nucleic acid extraction—especially in plate-based workflows—can lead to microbial signal carryover between adjacent wells, distorting microbial profiles [94].

To mitigate these risks, robust quality control is essential. This includes the use of no-template controls, extraction blanks, and standardized mock microbial communities. Internal spike-in controls also help benchmark assay sensitivity and inter-run variability [95]. Bioinformatics pipelines are evolving to incorporate contamination-aware algorithms, improved taxonomic classifiers, and cloud-based tools (e.g., IDSeq, One Codex) that enhance diagnostic precision and facilitate clinical translation. In summary, although mNGS offers unparalleled breadth in pathogen detection, its technical limitations—especially host DNA overabundance, contamination risks, and sensitivity issues—must be addressed through innovations in sample processing, depletion chemistry, and computational filtering to support reliable clinical application.

### 4.2. Bioinformatics Standardization and Interpretive Challenges

The diagnostic utility of mNGS depends not only on sequencing technologies but also—critically—on the robustness, transparency, and standardization of bioinformatics pipelines. Currently, the lack of harmonized interpretive criteria—including taxonomic classification thresholds, AMR gene annotation parameters, and clinical relevance filters—introduces significant inter-platform variability and undermines diagnostic consistency. A major barrier to clinical adoption lies in the heterogeneity of computational tools and analytical workflows across laboratories. Most bioinformatics pipelines employ either alignment-based methods (e.g., BLAST v2.14.0, BWA) or k-mer-based classifiers (e.g., Kraken2, Centrifuge) for taxonomic assignment. Alignment-based tools offer high specificity and contextual accuracy but are computationally intensive and prone to missed detection of highly divergent or low-abundance organisms [96]. In contrast, k-mer-based algorithms are computationally efficient and more sensitive to low-level microbial signals, yet they are more susceptible to false positives due to shared k-mer profiles among closely related taxa [30]. This sensitivity–specificity tradeoff directly impacts clinical interpretation—especially in low-biomass samples such as plasma, CSF, or BALF, where the microbial signal is often obscured by host DNA and environmental contaminants [8].

Further complicating interpretation is the variability in classifier thresholds, database completeness, and read-filtering algorithms, all of which can skew sensitivity and specificity across platforms. Almeida et al. [97] demonstrated that tool performance varies significantly with reference database structure, while Escobar-Zepeda et al. [98] highlighted how differing AMR gene databases and annotation criteria yield inconsistent resistome profiles, potentially undermining antimicrobial stewardship.

Several platforms aim to streamline microbial profiling, each with strengths and limitations. Kraken2 uses a k-mer approach for rapid classification but is heavily dependent on the completeness of its underlying database [30]. IDSeq, a cloud-based tool optimized for resource-limited settings, incorporates integrated contamination filtering, though performance may fluctuate with sequencing depth and organism type [29]. One Codex, a commercial platform with curated microbial reference libraries, offers user-friendly dashboards but uses proprietary, non-transparent algorithms, limiting external validation and compliance with regulatory standards [99]. This “black box” approach presents a significant barrier to the independent verification, standardization, and quality assurance required for widespread adoption in accredited clinical laboratories. Without transparent bioinformatic algorithms and clearly defined interpretive thresholds, mNGS remains constrained as a research tool rather than a validated diagnostic assay ready for clinical implementation. The lack of regulatory oversight for proprietary software further complicates efforts to compare results across platforms or institutions, limiting reproducibility and broader clinical uptake. As highlighted by Chiu and Miller [5,7], the absence of standardized analytical validation frameworks and public access to underlying pipelines poses a major hurdle in translating mNGS from bench to bedside.

Comparative studies underscore the clinical consequences of such variability. Besser et al. [100] demonstrated that changing a single read threshold could convert a positive sample to negative in polymicrobial or low-biomass contexts. A Swiss-wide inter-laboratory ring trial similarly revealed wide discrepancies in viral pathogen detection, driven by differences in genome coverage thresholds and filtering criteria [101]. These findings emphasize the urgent need for standardized, benchmarked pipelines to support clinical confidence.

Resistance gene detection presents additional complexities. Tools such as CARD, ResFinder, and MEGARes are widely used, but differ in curation methods, gene nomenclature, and database scope, often leading to conflicting resistance profiles from the same dataset [70,71]. More critically, there is no consensus on how to interpret detected resistance genes in terms of clinical actionability—especially when gene expression levels, horizontal transfer potential, and resistance mechanisms remain undefined.

To address these gaps, professional societies and regulatory bodies must lead the development of consensus-driven standards for bioinformatics interpretation. Organizations such as the CLSI and IDSA should define minimum read thresholds, genome coverage requirements, taxonomic confidence scoring, AMR annotation guidelines, and standardized result reporting formats. Without such frameworks, mNGS risks remaining a research tool rather than a validated diagnostic assay suitable for clinical decision-making.

### 4.3. Clinical Integration and Diagnostic Stewardship

While mNGS is increasingly recognized for its diagnostic potential, its integration into routine clinical workflows remains hindered by interpretive complexity and variable clinician familiarity. A recurring challenge arises when results report low-abundance reads or multiple microbial taxa, creating uncertainty about whether the findings reflect true infection, colonization, or contamination [4]. Without standardized interpretive frameworks and clinical decision pathways, such ambiguities may reduce the utility and actionability of mNGS results.

Evidence suggests that interdisciplinary diagnostic stewardship—involving infectious disease specialists, clinical microbiologists, and pharmacists—can significantly enhance the clinical impact of mNGS. A retrospective analysis conducted at two U.S. academic medical centers demonstrated that integrating mNGS interpretation into antimicrobial stewardship rounds improved diagnostic confidence, optimized test utilization, and positively influenced therapeutic decisions [102].

Embedding mNGS results into electronic health records (EHRs)—especially when accompanied by interactive decision-support tools—further facilitates timely and appropriate prescribing. One study showed that such integration enabled early de-escalation of broad-spectrum antibiotics in over 30% of cases, without compromising clinical outcomes [48,103]. These findings highlight the value of pairing high-resolution molecular data with structured, clinician-facing decision support.

The timing and context of result delivery also play a pivotal role. A Swiss-wide interpretive trial revealed that mNGS results were most impactful when reviewed collaboratively by laboratory professionals and treating physicians at the point of care, underscoring the importance of real-time consultation and shared diagnostic responsibility. Equally important is provider education. A commentary in Nature Reviews Genetics called for structured training programs targeting physicians, microbiologists, pharmacists, and stewardship personnel [5]. These initiatives should cover the technical principles of mNGS, limitations in low-biomass contexts, the interpretation of polymicrobial results, and the integration of findings into antimicrobial stewardship frameworks. Without this foundational knowledge, clinicians may either overinterpret incidental findings or undervalue clinically relevant results, reducing diagnostic reliability and patient benefit.

Although the promise of mNGS is substantial, several expert reviews advise cautious, evidence-based implementation. Chiu and Miller [5] emphasized the current lack of standardized interpretive criteria, limited prospective validation studies, and uncertain cost-effectiveness as key barriers to widespread use. Similarly, Simner et al. [4] raised concerns regarding inter-assay variability, ambiguous readouts, and interpretive subjectivity—all of which limit reproducibility. Both reviews recommend that mNGS be deployed selectively, ideally in well-resourced settings with strong diagnostic infrastructure, interdisciplinary stewardship teams, and the capacity for expert genomic data interpretation [10,51]. These perspectives underscore the importance of prudent implementation, guided by robust clinical validation and clear consensus frameworks, to ensure that mNGS fulfills its potential as a transformative diagnostic tool in infectious disease care.

### 4.4. Cost, Turnaround Time, and Health Economics

Despite the growing enthusiasm for mNGS in infectious disease diagnostics, its broad clinical implementation remains constrained by cost, turnaround time (TAT), and uncertain health economic value—particularly in low- and middle-income countries (LMICs). While sequencing costs have steadily declined, the total expenditure associated with mNGS remains substantial. Reported per-sample costs range from approximately USD 100 for Illumina-based workflows to over USD 300 for nanopore sequencing-based respiratory metagenomics, depending on the platform, sequencing depth, and regional infrastructure [104]. These figures often exclude hidden costs such as labor, reagents, computational resources, data storage, and regulatory compliance—all of which can markedly increase the overall price in clinical settings.

TAT is another key determinant of mNGS’s clinical impact. While conventional workflows yield results in 24–72 h, this is still slower than most rapid multiplex PCR assays. Nonetheless, technological advancements are accelerating diagnostic speed. In a prospective ICU study at Guy’s and St Thomas’ Hospital in London, same-day results were achieved in 86% of respiratory samples using nanopore sequencing, with a median TAT of 6.7 h. Notably, mNGS results influenced antimicrobial decision-making in nearly half of cases, demonstrating that rapid implementation can deliver real-time therapeutic benefit [105].

However, it is essential to recognize that mNGS detects nucleic acids, not necessarily viable pathogens. Microbial DNA may originate from commensals, colonizers, or non-viable organisms—particularly in samples from non-sterile sites like the respiratory tract, gut, or skin. This creates a risk of overdiagnosis and overtreatment, especially in immunocompromised or critically ill patients. Furthermore, microbial cfDNA may persist following clinical resolution or antibiotic therapy, complicating interpretation. Accurate diagnosis, therefore, requires context-aware interpretation that incorporates clinical presentation, radiological findings, host biomarkers, and traditional microbiology results [5,8]. These limitations, if unaccounted for, can lead to misdiagnosis, unnecessary antimicrobial exposure, or erosion of clinician trust in mNGS. To mitigate these risks, standardized interpretive frameworks and diagnostic stewardship are essential to distinguish true infection from colonization or contamination [46].

Despite these interpretive challenges, mNGS may provide downstream economic benefits by improving diagnostic yield, guiding early targeted therapy, and reducing ICU stays. However, robust cost-effectiveness analyses are still lacking. In one study by Sutton et al. [106], adding plasma microbial cfDNA sequencing to conventional diagnostics in immunocompromised patients improved diagnostic yield and time to appropriate therapy, but long-term cost-effectiveness metrics—such as cost per quality-adjusted life year (QALY)—were not evaluated.

In the context of bloodstream infections and sepsis, mNGS has shown diagnostic advantages. Zhou et al. [44] reported that mNGS identified causative pathogens in 65.7% of suspected sepsis cases, compared to only 12.1% with standard blood cultures. Moreover, results were returned within 24 h, significantly faster than traditional methods (>45 h). Still, the study emphasized the need for formal budget-impact and cost–benefit analyses before broader implementation can be justified. Ultimately, while mNGS offers substantial clinical value, its sustainable integration into health systems—particularly in resource-constrained settings—will depend on further reductions in cost, accelerated TATs, and rigorous health economic evaluations that demonstrate value relative to existing diagnostic approaches.

### 4.5. Ethical, Legal, and Privacy Considerations

The clinical and public health application of mNGS raises a complex array of ethical, legal, and privacy concerns, largely stemming from its dual capacity to capture both microbial and host-derived genomic information. This breadth of detection introduces risks related to incidental findings, patient autonomy, data governance, and equitable access. A key ethical challenge involves the unintentional discovery of human genetic variants—such as those linked to hereditary cancer syndromes, primary immunodeficiencies, or pharmacogenomic traits—during routine mNGS testing. Jelsig et al. [107] emphasized the necessity of pre-test informed consent frameworks that clearly communicate the possibility of incidental findings and allow patients to specify whether and how such information should be disclosed. Without explicit consent mechanisms, there is a risk of breaching patient autonomy and undermining trust in genomic diagnostics.

Beyond incidental discoveries, concerns over data ownership and privacy remain paramount. Even de-identified genomic datasets can be vulnerable to re-identification through cross-referencing with external databases or machine learning algorithms. Azencott [108] underscored the need for robust safeguards—such as data encryption, restricted access protocols, and transparent governance structures—to prevent misuse of genetic information, particularly in contexts such as insurance discrimination, employment bias, or unauthorized data sharing.

These risks are magnified in countries lacking comprehensive genomic data protection laws. For example, while the U.S. Genetic Information Nondiscrimination Act (GINA) prohibits genetic discrimination in health insurance and employment, it does not extend protections to life insurance or long-term care. Similarly, while the European Union’s General Data Protection Regulation (GDPR) offers stringent privacy standards, its implementation and enforcement vary across member states, particularly regarding clinical genomics. Such legal inconsistencies can create regulatory uncertainty, limit public confidence, and impede patient participation in mNGS-based diagnostics and research.

Global disparities in access to sequencing technologies further exacerbate ethical tensions. Chiang [109] noted that high-throughput sequencing infrastructure is predominantly concentrated in high-income countries, limiting the ability of LMICs to participate in genomic surveillance, research, and clinical benefit. Without targeted investments in infrastructure and capacity-building, mNGS may widen rather than bridge existing global health inequities.

Public health applications of mNGS—such as outbreak surveillance and AMR tracking—raise additional ethical considerations. While the timely sharing of sequencing data is vital for controlling epidemics and informing global responses, it must be balanced against the rights of individuals and communities. Johnson et al. [110] argued that mNGS-based public health interventions must be governed by ethical frameworks that uphold confidentiality, community engagement, and transparent data-sharing agreements, especially during emergencies.

In summary, the clinical and public health integration of mNGS must be accompanied by clear ethical guidelines, legal protections, and privacy-preserving technologies. These safeguards are essential not only for protecting patient rights but also for fostering public trust, international cooperation, and equitable benefit-sharing as mNGS becomes increasingly embedded in infectious disease diagnostics and global health surveillance.

### 4.6. Regulatory and Reimbursement Landscape

Despite the growing promise of mNGS in infectious disease diagnostics, its widespread clinical adoption remains hampered by regulatory ambiguity and inadequate reimbursement pathways. These systemic barriers contribute to variability in test implementation, hinder standardization, and limit accessibility—especially outside of academic or specialized centers. In the United States, most mNGS assays for infectious diseases are currently offered under CLIA-certified frameworks as laboratory-developed tests (LDTs). However, to date, no mNGS platform has received full approval from the U.S. Food and Drug Administration (FDA) for infectious disease applications. As highlighted by Chiu et al. [111], this regulatory gap allows significant heterogeneity in assay validation, quality control, and result reporting, ultimately constraining inter-laboratory reproducibility and regulatory confidence.

Reimbursement presents a parallel challenge. In the U.S. healthcare system, CPT codes, established by the American Medical Association (AMA), are essential for billing and reimbursement of diagnostic services. Yet, no dedicated CPT codes currently exist for mNGS-based infectious disease testing, resulting in administrative uncertainty. Tests are often submitted under non-specific molecular codes or pieced together using multiple components—an approach that leads to undercoding, reimbursement delays, or outright denials [112].

Payers—including Medicare and commercial insurers—require robust evidence of clinical utility, cost-effectiveness, and analytical validity before approving reimbursement. In the absence of standardized validation protocols and real-world outcome data, mNGS claims are often evaluated on a case-by-case basis, with frequent denials. [113]. A Medicare claims analysis by Lee et al. [114] found that more than 23% of sequencing-related submissions in oncology were denied between 2016 and 2021, a pattern likely mirrored in infectious disease diagnostics given similar evidentiary gaps.

To address these barriers, large-scale, prospective clinical trials are essential. Demonstrating that mNGS leads to earlier pathogen detection, reduced empirical antibiotic use, decreased ICU stays, and improved outcomes is critical to establishing a compelling health economic and regulatory rationale for broader use. Simultaneously, professional societies and regulatory agencies must define performance benchmarks for clinical mNGS. These should include minimum analytical sensitivity and specificity thresholds, contamination controls, read depth requirements, standardized reporting formats, and criteria for actionable findings [4]. Such benchmarks are necessary not only for validation and regulatory approval but also for consistent payer assessments and laboratory accreditation.

Efforts toward regulatory harmonization are also underway in Europe. The European Society of Clinical Microbiology and Infectious Diseases (ESCMID), through its ESGMD working group, has developed consensus quality criteria for clinical metagenomics to support future Conformité Européenne (CE) marking and facilitate standardized adoption across EU member states [115]. These frameworks aim to streamline mNGS approval and ensure regulatory consistency across national borders.

Until such regulatory clarity and reimbursement infrastructure are established, mNGS will likely remain concentrated within academic centers and reference laboratories, limiting its scalability and equity. Cross-sector collaboration—among clinicians, laboratory scientists, payers, policymakers, and industry partners—is essential to transition mNGS from an investigational tool to a reimbursable, validated diagnostic modality in infectious disease care.

### 4.7. Future Innovations and Research Priorities

The ongoing evolution of mNGS is fueled by a range of technological and methodological innovations aimed at enhancing diagnostic speed, accuracy, clinical interpretability, and global accessibility. These advances are essential for overcoming current translational bottlenecks and realizing the full potential of mNGS as a frontline diagnostic tool. One key area of innovation is the development of portable, real-time sequencing platforms, particularly those based on nanopore technology. These devices enable rapid, decentralized pathogen detection, which is particularly valuable in emergency settings, resource-limited environments, and point-of-care applications. Charalampous et al. [28] demonstrated that optimized nanopore workflows could identify respiratory pathogens directly from clinical specimens in under six hours, showing strong concordance with culture-based methods. This rapid turnaround provides a critical advantage for time-sensitive clinical decision-making, such as in the intensive care unit or during outbreak response.

AI and ML are also poised to revolutionize mNGS bioinformatics. These tools can automate complex analytical tasks—including taxonomic classification, resistance gene detection, contamination filtering, and host–pathogen interaction modeling. A study by Olatunji et al. [116] showed that deep learning classifiers significantly outperformed conventional tools in detecting AMR genes, with improvements in both accuracy and speed. As these AI-enhanced systems mature, they may reduce reliance on highly specialized personnel, support real-time analysis, and improve diagnostic consistency across platforms.

Another promising development is the integration of multi-omics approaches. By combining metagenomics with metatranscriptomics, proteomics, and host transcriptomics, clinicians can gain a more comprehensive view of infectious processes. This systems-level approach improves the ability to differentiate between active infection, colonization, and sterile inflammation. For example, Blauwkamp et al. [46] reported that coupling microbial cfDNA sequencing with host immune response markers significantly improved diagnostic performance in immunocompromised patients—illustrating the potential of integrated diagnostics.

Global health equity remains a critical priority. Without focused investment, mNGS may remain restricted to high-resource settings. Innovations such as lyophilized reagents, solar-powered sequencers, and simplified workflows are helping bridge this gap. During the West African Ebola outbreak, Quick et al. [13] successfully deployed portable nanopore sequencers in the field, enabling real-time genomic surveillance in austere environments—a proof of concept for mNGS in global outbreak response and One Health applications.

Despite these technical gains, robust clinical validation is essential to transition mNGS from research to routine care. Large, multicenter, prospective trials are needed to demonstrate its impact on patient outcomes, antibiotic stewardship, and healthcare costs. One notable example is the MATESHIP trial, conducted across 20 ICUs in China, which is evaluating mNGS-guided care in immunocompromised patients with severe pneumonia. Endpoints include time to targeted therapy, duration of ICU stay, antimicrobial consumption, and mortality [117].

In parallel, global data-sharing initiatives are gaining momentum to improve outbreak preparedness and AMR surveillance. Aamot et al. [115], on behalf of the ESCMID Study Group for Public Health Microbiology (ESGPHM), advocated for federated, privacy-preserving data networks that support secure, timely exchange of genomic data across jurisdictions. Such frameworks are essential not only for real-time threat detection but also for equitable global response coordination.

In brief, the future of mNGS will be defined by miniaturized, field-ready platforms, AI-enhanced analytics, multi-omics diagnostics, global data collaboration, and clinical validation through multicenter trials. These innovations collectively hold the potential to transform infectious disease diagnostics and reinforce global health security. To provide a consolidated overview of the current barriers and strategic solutions for the clinical adoption of mNGS, Table 3 summarizes key translational challenges across technical, interpretive, economic, and ethical dimensions, alongside proposed innovations and supporting references.

To support the clinical integration of mNGS, numerous prospective clinical trials (Table 4) have been completed or are currently ongoing across a range of healthcare settings. Table 3 provides a consolidated summary of key mNGS-based trials, outlining their study design, target populations, infectious disease indications, diagnostic modalities, and primary clinical endpoints. Collectively, these studies are designed to evaluate the diagnostic utility, therapeutic impact, and clinical outcomes associated with mNGS in real-world practice. Notably, large-scale randomized controlled trials (RCTs) such as MATESHIP and GRAIDS are assessing the comparative effectiveness of mNGS-guided management versus conventional diagnostics in high-risk patient populations, including those who are immunocompromised or critically ill. These trials are critical for validating mNGS as a standard-of-care diagnostic tool and informing evidence-based treatment protocols.

Other initiatives, including the DISQVER and REMEDID studies, explore the value of mNGS as a complementary adjunct to traditional microbiological testing. These observational and interventional trials are instrumental in identifying use cases where mNGS enhances diagnostic yield, expedites antimicrobial optimization, or improves patient-centered outcomes such as length of ICU stay, time to appropriate therapy, and mortality rates. By generating high-quality clinical and health economic data, these studies are paving the way for regulatory approval, reimbursement framework development, and the broader adoption of mNGS technologies in routine infectious disease diagnostics.

## 5. Conclusions

Metagenomic next-generation sequencing (mNGS) is transforming the landscape of infectious disease diagnostics. By enabling unbiased, comprehensive pathogen detection—without the need for prior assumptions—mNGS serves as a powerful adjunct to conventional culture and targeted molecular assays, particularly in diagnostically challenging or atypical presentations. As detailed throughout this review, mNGS is capable of identifying fastidious, novel, and polymicrobial infections, facilitating AMR profiling, and supporting real-time outbreak surveillance. These attributes are particularly critical in immunocompromised and critically ill patients, where diagnostic speed and accuracy can significantly influence clinical outcomes. However, the clinical translation of mNGS remains a complex endeavor. Technical limitations—including host DNA overrepresentation, low microbial biomass, and background contamination—continue to impact analytical sensitivity and specificity. In parallel, bioinformatics inconsistencies stemming from non-standardized pipelines, variable classification thresholds, and heterogeneous resistance gene databases hinder result reproducibility and clinical interpretation. Furthermore, ethical and legal challenges, such as the management of incidental host genome findings and data privacy concerns, complicate implementation—especially in the context of global health inequities and fragile public trust. Financial and regulatory obstacles also remain substantial. The lack of FDA-approved mNGS assays, variable payer reimbursement policies, and high implementation costs collectively restrict access, particularly in LMICs. Despite these hurdles, large-scale clinical trials—including MATESHIP, GRAIDS, DISQVER, and NGS-CAP—are generating high-quality evidence on the diagnostic yield, clinical utility, and therapeutic value of mNGS-guided care. These studies are essential for informing policy development, regulatory frameworks, and reimbursement decisions. Looking ahead, the future of mNGS will be shaped by technological innovation and system-level integration. Advances in portable sequencing platforms, artificial intelligence-enhanced analytics, and multi-omics integration are poised to accelerate diagnostic turnaround time, improve interpretability, and support broader clinical use. Simultaneously, the establishment of regulatory standards, harmonized reporting guidelines, and equitable data-sharing frameworks will be pivotal to widespread adoption. Simplified workflows and reduced costs will be critical for ensuring scalability and access in resource-limited settings. In conclusion, mNGS holds extraordinary promise as a next-generation diagnostic modality with the capacity to reshape both clinical microbiology and infectious disease management. However, its optimal role is as a complementary tool, enhancing—rather than replacing—traditional diagnostics by enabling broader pathogen detection, accelerating therapeutic decision-making, and supporting precision medicine approaches. When embedded within a multidisciplinary, stewardship-oriented diagnostic strategy, mNGS is positioned to enhance patient care, reinforce antimicrobial stewardship, and strengthen global health preparedness. As technological, clinical, and policy ecosystems continue to evolve in concert, mNGS is on track to become a cornerstone of infectious disease diagnostics in the 21st century.

## Figures and Tables

**Table 1:** Comparative overview of NGS platforms in clinical microbiology.

NGS Modality	Sequencing Scope	Advantages	Limitations	Clinical Use Cases
WGS [22]	Complete genome (from cultured isolate)	High resolution; AMR/virulence detection; outbreak tracing	Requires culture; slower turnaround	Bacterial typing; resistance surveillance
mNGS [8,9]	All DNA/RNA in sample (unbiased)	Detects unknown/rare pathogens; no culture needed	High host DNA background; expensive; complex analysis	Meningitis; sepsis of unknown origin; rare pathogens
Targeted NGS Panels [28]	Predefined microbial/resistance genes	Faster; lower cost; easier interpretation	Limited to panel design; misses unexpected targets	Syndromic panels (respiratory, GI, sepsis)
Long-read Sequencing (ONT/PacBio) [13,34]	Long fragments (up to >10 kb)	Portable; real-time sequencing; resolves complex genomic structures	Higher error rates; infrastructure variable	Point-of-care outbreak diagnostics; TB genomics
Transcriptomics/Single-cell RNA-seq [31,33]	Host RNA expression	Host response profiling; immune status insights	Emerging; complex interpretation; mostly research stage	Disease severity prediction; host–pathogen studies
Automated Platforms [46]	Integrated sample-to-answer systems	Same-day results; minimal manual input	Initial setup cost; platform-dependent limitations	Rapid diagnostics in hospital labs
Cloud-based Pipelines [29,30]	Data analysis only	Removes need for in-house bioinformatics; scalable	Dependent on internet; privacy/security concerns	Clinical metagenomics (low-resource labs)

With the foundational sequencing technologies, analytical modalities, and bioinformatic pipelines now introduced, the focus shifts from methodological underpinnings to real-world clinical implementation. The following section explores how next-generation sequencing—particularly metagenomic approaches—has been operationalized across a range of infectious disease settings. From diagnostic clarification in undifferentiated infections to therapeutic guidance and outbreak investigation, NGS is increasingly shaping clinical decision-making. This transition from the bench to the bedside highlights the translational value of NGS and its expanding role in enhancing patient outcomes, antimicrobial stewardship, and public health surveillance.

**Table 2:** Summary of real-world mNGS applications across infectious syndromes.

Study	Clinical Syndrome	Sample Type	Key Findings	Clinical Impact
Xiang et al. [82]	Post-cardiac surgery pneumonia	BALF	98.2% diagnostic yield vs. 58.4% by culture	Reduced mechanical ventilation duration, improved SOFA scores, shorter ICU stays
Shi et al. [83]	Vertebral osteomyelitis	Biopsy	77.8% detection by mNGS vs. 27.2% by culture; identified anaerobes and polymicrobial infections	High specificity (90.3%); effective despite prior antibiotics
Huang et al. [84]	PJI	Synovial Fluid	89% sensitivity, 95% specificity	Enabled antibiotic de-escalation without compromising efficacy
Shi et al. [85]	PJI	Synovial Fluid	89.1% sensitivity, 94.7% specificity; detected fastidious organisms	Supported tailored therapy and improved diagnostic confidence
Zhang et al. [86]	Systemic infections/sepsis	Plasma (cfDNA)	74.4% mNGS detection vs. 12.1% by culture	70.3% had antimicrobial therapy adjusted; earlier sampling linked to shorter hospital stay

**Table 3:** Key translational challenges and proposed solutions for clinical implementation of mNGS in infectious disease diagnostics.

Challenge	Description	Potential Solutions	References
High host DNA background	Over 90% of reads in plasma/CSF may be host-derived, masking microbial signals	Host depletion via saponin lysis, DNase digestion, or CRISPR-Cas9 (DASH)	[89,90]
Environmental contamination (“kitome”)	Reagent-based or lab-introduced contaminants skew microbial profiles	Inclusion of no-template/extraction blanks; contamination-aware bioinformatics	[94,95]
Bioinformatics inconsistency	Non-standardized taxonomic classification and AMR annotation lead to variable results	Harmonized pipelines, curated databases, confidence thresholds, consensus guidelines	[4,70,71]
Clinical interpretation uncertainty	Difficulty distinguishing colonization, contamination, or infection in low-abundance reads	Expert stewardship teams, integrated EHR tools, multidisciplinary training	[5,78,103]
Cost and turnaround time	mNGS costs USD 100–USD 300/sample; results take 24–72 h; delays care in some settings	Streamlined nanopore workflows, automation, targeted panels	[105,106]
Regulatory and reimbursement barriers	Lack of FDA approval and CPT codes; insurance denials common	Prospective validation trials; CPT reform; CLSI/IDSA performance guidelines	[4,115]
Ethical, legal, and privacy issues	Incidental human findings, re-identification risk, genomic discrimination	Transparent consent, data encryption, privacy laws, governance frameworks	[108,110]
Global equity and access	High-income countries dominate infrastructure and data; LMICs underrepresented	Capacity-building, portable devices, federated data sharing	[13,115]

**Table 4:** Summary of major clinical trials on mNGS in infectious disease diagnosis.

Trial Name	Study Design	Population	Infection Type	Intervention Strategy	Primary Outcome(s)	Reference
MATESHIP Trial	Randomized Controlled Trial (RCT)	Immunocompromised ICU patients	Severe Community-Acquired Pneumonia	mNGS-guided therapy vs. standard culture	Antibiotic duration, ICU LOS, mortality	[117]
DISQVER Trial	Prospective Observational Study	Septic patients with suspected infection	Bloodstream and respiratory infections	cfDNA mNGS vs. conventional blood cultures	Pathogen ID rate, time to targeted therapy	[118]
REMEDID Study	Multicenter Observational Study	Immunosuppressed hematology patients	Hematologic febrile neutropenia	BAL fluid mNGS for pathogen detection	Diagnostic yield, antifungal escalation	[119]
GRAIDS Trial	Multicenter Randomized Trial	Critically ill ICU patients	Pulmonary and systemic infections	mNGS plus EHR decision support	Antibiotic use, time to diagnosis	[120]
Karius Prospective Study	Prospective Multicenter Cohort	Hospitalized adults with suspected infection	Broad-spectrum infections	cfDNA plasma NGS (Karius test)	Diagnostic yield, time to intervention	[46]
NGS-CAP (China)	Prospective Diagnostic Evaluation	Severe CAP patients in ICU	CAP or HAP (non-responders)	Illumina mNGS-based workflow	Detection rate, concordance with cultures	[121]
Illumina IDbyDNA Evaluation	Comparative Clinical Validation	Patients with suspected CNS infections	CNS infections (encephalitis, meningitis)	IDbyDNA PathoScope NGS platform	Sensitivity, specificity, turnaround time	[8]
NIDP-Fungal mNGS Trial	Prospective Observational Trial	Patients with suspected fungal pneumonia	Invasive fungal infections (IFIs)	Fungal mNGS assay vs. conventional tests	Positive detection rate, clinical impact	[122]

## Data Availability

Not applicable. No new data were created or analyzed in this study.

## Abbreviations

mNGS	Metagenomic Next-Generation Sequencing
AMR	Antimicrobial Resistance
WGS	Whole Genome Sequencing
cfDNA	Cell-Free DNA
ICU	Intensive Care Unit
CAP	Community-Acquired Pneumonia
EHRs	Electronic Health Records
AI	Artificial Intelligence
ML	Machine Learning
BALF	Bronchoalveolar Lavage fluid
RCT	Randomized Controlled Trial
FDA	Food and Drug Administration
PCR	Polymerase Chain Reaction
ESCMID	European Society of Clinical Microbiology and Infectious Diseases
GLASS	Global Antimicrobial Resistance Surveillance System
SNP	Single Nucleotide Polymorphism
RNA	Ribonucleic Acid
DNA	Deoxyribonucleic Acid
IDSA	Infectious Diseases Society of America
CLSI	Clinical and Laboratory Standards Institute
NGS	Next-Generation Sequencing
HAP	Hospital-Acquired Pneumonia
EU	European Union
